# Periodontal knowledge and clinical attitudes of family physicians in Turkey: a cross-sectional survey

**DOI:** 10.1186/s12875-026-03324-3

**Published:** 2026-04-22

**Authors:** Meryem Hüsna Akkaya, Emre Yaprak, Ahmet Doğukan Gündoğdu, Yasemin Turan Akansel, Tuncay Müge Alvur

**Affiliations:** 1 Istanbul Public Health Center, Ministry of Health, Istanbul, Turkey; 2 Department of Periodontology, Faculty of Dentistry, Kocaeli University, Kocaeli, Turkey; 3 Department of Family Medicine, Faculty of Medicine, Kocaeli University, Kocaeli, Turkey

**Keywords:** Periodontal disease, Family physicians, Oral health awareness, Preventive dentistry, Interdisciplinary referral, Antimicrobial stewardship, KAP study, Cross-sectional study

## Abstract

**Background:**

Periodontitis is one of the most prevalent non-communicable chronic diseases globally, and its early recognition is critical for preventive healthcare. Primary care providers, particularly family physicians, occupy a strategically advantageous position for early detection of oral diseases and timely referral for professional dental care. The primary aim of this study was to explore whether differences exist in periodontal knowledge and clinical attitudes between certified family physicians (CFPs) and family medicine specialists/residents (FMSs) compared with dentists, with the latter serving as a professionally trained reference group.

**Methods:**

A descriptive, cross-sectional survey was conducted in Turkey between June and August 2020. Participants were recruited through convenience sampling via professional social media networks (WhatsApp and Facebook groups). A 42-item online questionnaire was distributed to dentists, CFPs, and FMSs via Google Forms. Of 818 initiated responses, 808 met the inclusion criteria (completion rate: 98.8%). Internal consistency for the knowledge domain was assessed using the Kuder–Richardson Formula 20 (KR-20 = 0.74). Statistical analyses employed chi-square tests with Cramér’s V effect sizes, Kruskal–Wallis tests, and multivariable logistic regression adjusting for age, sex, and professional experience, with Bonferroni-corrected post-hoc comparisons (significance level *p* < 0.05).

**Results:**

The final cohort consisted of 377 dentists (46.7%), 227 CFPs (28.1%), and 204 FMSs (25.2%). Family physicians demonstrated significantly lower knowledge than dentists across all assessed domains (*p* < 0.001). The majority of family physicians (69.2% of CFPs, 71.6% of FMSs) erroneously identified toothache as a primary sign of periodontitis. Only 13.7% of CFPs and 13.2% of FMSs correctly identified all three drug classes associated with gingival enlargement, versus 63.1% of dentists. Awareness of adverse pregnancy outcomes linked to periodontitis was low among CFPs (52.5%) and FMSs (38.7%). Direct referral of suspected dental infections without prior antibiotic or antiseptic prescription was observed in only 24.2% of CFPs and 8.3% of FMSs. After adjustment for demographic confounders, professional group remained a significant independent predictor of knowledge outcomes (adjusted OR range: 0.04–0.38 for family physicians vs. dentists as reference; all *p* < 0.001), confirming that the observed differences were not attributable to age, sex, or experience.

**Conclusions:**

These findings suggest that family physicians in Turkey may have notable gaps in periodontal knowledge across multiple domains. While these results should be interpreted with caution given the convenience sampling methodology and cross-sectional design, they are consistent with comparable international studies. Further research employing probability-based sampling and interventional designs is warranted to determine whether targeted educational initiatives could improve interdisciplinary oral–systemic care.

**Supplementary Information:**

The online version contains supplementary material available at 10.1186/s12875-026-03324-3.

## Background

Oral health is an indispensable component of overall well-being that profoundly influences the qualitative standards of human life [1]. Although oral pathologies are largely preventable, severe periodontitis remains the sixth most prevalent disease globally according to the Global Burden of Disease (GBD) data, affecting approximately 743 million individuals and constituting a formidable public health challenge [2, 3]. Periodontitis is a chronic, multifactorial inflammatory disease associated with dysbiotic plaque biofilms, characterized by the progressive destruction of the tooth-supporting apparatus, encompassing the periodontal ligament, alveolar bone, and root cementum [4, 5]. Periodontitis contributes to tooth loss and masticatory dysfunction and has been associated with major non-communicable diseases through shared modifiable risk factors. Among these, a bidirectional relationship is most clearly established with diabetes mellitus [6, 7]. The European Federation of Periodontology (EFP) S3-level clinical practice guideline emphasizes the importance of early detection and systematic management of periodontitis within an integrated healthcare framework [8]. The global burden of periodontal disease, in terms of the absolute number of affected individuals, increased substantially between 1990 and 2010, largely driven by population ageing, rising prevalence of metabolic risk factors, and persistent socioeconomic disparities in access to healthcare [3, 7, 9, 10].

As primary healthcare providers, family physicians encounter patients far more frequently than dental professionals during routine screenings and chronic disease management; therefore, they are uniquely positioned to identify periodontal conditions and bridge the gap between oral and systemic health [11]. This dynamic places family physicians in a highly advantageous position to facilitate the early detection of oral diseases, address modifiable risk behaviors, and ensure timely referral of patients to dental professionals. Nevertheless, despite high levels of awareness regarding the bidirectional relationship between diabetes and periodontitis, primary care physicians have incorporated periodontal assessment into routine clinical practice only to a limited extent; this suggests the persistence of structural and interdisciplinary gaps between medical and dental care delivery systems [6, 12, 13].

Turkey’s healthcare system assigns family physicians a central gatekeeping role in primary care. Under the Family Medicine Practice system (Aile Hekimliği Uygulaması) each registered citizen is assigned a family physician who serves as the primary point of contact for all health concerns. Despite this broad mandate, the standard medical curriculum in Turkey allocates minimal didactic time to oral health topics. A prior Turkish study assessed general oral health knowledge among family medicine residents but did not specifically evaluate periodontal health using a multi-domain validated instrument, to our knowledge [14]. This gap, combined with the absence of standardized oral health training modules in Turkish medical education, provides a clear rationale for the present investigation.

While existing literature encompasses KAP studies evaluating oral health awareness of medical practitioners across various global regions, including India [15], France [16], Hong Kong [17], Iran [18], and China [19], precise data focusing exclusively on the proficiency and clinical referral attitudes of family physicians regarding periodontal health remain sparse. Within this context, the present study was designed to explore whether family physicians in Turkey exhibit differences in periodontal knowledge and clinical attitudes compared with dentists. Dentists were included not as equivalent peers but as a professionally trained reference group, serving as a benchmark for the expected standard of periodontal knowledge. This design, consistent with previous KAP studies in the field [15, 16], allows quantification of the magnitude of knowledge gaps in family physicians relative to practitioners whose training explicitly covers periodontal disease. We acknowledge that this comparison reflects differences in professional training rather than individual competence, and results should be interpreted accordingly.

## Methods

### Study design and ethical approval

This research was designed as a descriptive, cross-sectional survey conducted in Turkey between June and August 2020. The study protocol was reviewed and approved by the Non-Interventional Clinical Research Ethics Committee of Kocaeli University (Approval No: GOKAEK-2020/78, 12 March 2020), in full accordance with the principles of the Declaration of Helsinki (2013 revision). Digital informed consent was obtained from all participants prior to survey initiation.

### Study population and sampling

The target population comprised three professional groups practicing in Turkey: (1) certified family physicians (CFPs), general practitioners holding a non-specialist certification in family medicine; (2) family medicine specialists and residents (FMSs), physicians in formal specialty training or holding a specialty degree in family medicine; and (3) dentists, who served as the reference group. The estimated target population was approximately 30,000 individuals. Sample size was calculated based on proportions reported in a comparable study [15] and a pre-test pilot.

A convenience sampling strategy was employed due to the nationwide scope of the study and the practical constraints imposed by the COVID-19 pandemic, which precluded in-person recruitment during the data collection period (June–August 2020). While this approach may introduce selection bias toward younger, digitally active practitioners, it has been widely utilized in healthcare KAP studies conducted during pandemic conditions and enables access to geographically dispersed professionals. The instrument was distributed as a Google Forms link via WhatsApp and Facebook professional networks.

The a priori sample size calculation indicated a minimum of approximately 380 respondents per group. While the dentist group (*n* = 377) closely approached this target, the CFP (*n* = 227) and FMS (*n* = 204) groups fell below the calculated minimum. This shortfall is attributable to the challenges of recruiting family physicians through social media channels during the COVID-19 pandemic. We acknowledge that the underachieved sample sizes in the family physician groups may reduce statistical power for detecting smaller effect sizes and limit the precision of point estimates. However, given that most observed differences were large in magnitude and statistically significant at *p* < 0.001, the risk of Type II error for the primary comparisons is considered low.

### Data collection instrument

A novel questionnaire was developed by the researchers specifically for the purposes of this study (Supplementary File 1). Content validity was established through expert review by two senior academic periodontologists (> 15 years of experience each) who independently assessed each item for relevance, clarity, and clinical accuracy. Items achieving consensus were retained. The questionnaire was subsequently pilot-tested with 25 family medicine residents and 15 recent dental graduates from Kocaeli University to assess comprehensibility and response burden (mean completion time: 12 ± 3 min). Internal consistency for the knowledge domain (Domain II) was assessed using the Kuder–Richardson Formula 20 (KR-20) for dichotomous items, yielding a coefficient of 0.74, indicating acceptable reliability. A full psychometric validation including test-retest reliability and factor analysis was not conducted, and this is acknowledged as a limitation.

The questionnaire comprised 42 items structured into three domains. Domain I (6 items) collected socio-demographic data: sex, age group (≤ 29 or ≥ 30 years), professional category, and years of clinical experience. Domain II (26 items; 17 multiple-choice and 9 multiple-response) assessed knowledge of periodontal disease definition; clinical signs and diagnostic criteria; systemic associations (diabetes, cardiovascular disease, rheumatoid arthritis); obstetric implications; drug-induced gingival changes; tobacco effects on periodontal clinical presentation; and antibiotic prescribing and preventive counselling attitudes. Domain III (10 items) assessed personal oral hygiene practices and self-reported periodontal status. Personal oral hygiene data (Domain III) were collected to explore whether practitioners’ own oral health behaviors correlate with their professional knowledge and counseling attitudes, consistent with the ‘physician health–practice style’ hypothesis documented in preventive medicine literature. This domain was not a primary outcome but was included as an exploratory variable. Self-perceived knowledge was assessed on a 0–10 visual numeric scale, categorized as low (0–3), moderate (4–6), and good (7–10).

### Statistical analysis

All data were exported from Google Forms to Microsoft Excel and analyzed using IBM SPSS Statistics (Version 20.0; IBM Corp., Armonk, NY, USA). Descriptive statistics are presented as frequencies and percentages for categorical variables, and as median (25th–75th percentile) for non-normally distributed continuous variables. Intergroup categorical comparisons were performed using the chi-square test, with Cramér’s V reported as a measure of effect size (interpreted as small ≥ 0.10, medium ≥ 0.30, large ≥ 0.50). The 95% confidence intervals for key proportions were calculated using the Wilson method. The Kruskal–Wallis test was applied for non-parametric continuous data and Student’s t-test for parametric comparisons. Post-hoc pairwise comparisons were conducted with Bonferroni correction.

To account for potential confounding by demographic variables, multivariable binary logistic regression analyses were performed for key outcome variables (correct identification of clinical signs, knowledge of systemic associations, appropriate referral behavior). Professional group was entered as the primary independent variable, with age group (≤ 29 vs. ≥30 years), sex, and years of clinical experience (≤ 5 vs. >5 years) included as covariates. Results are reported as adjusted odds ratios (aOR) with 95% confidence intervals in Supplementary Table S1. Statistical significance was set at *p* < 0.05.

### Use of artificial intelligence tools

The manuscript text was revised and refined with the assistance of an AI-based language model (Claude, Anthropic). The AI tool was used solely for language editing, structural improvement, and formatting purposes. All scientific content, data interpretation, conclusions, and intellectual contributions are entirely those of the authors. The authors take full responsibility for the accuracy and integrity of the work.

## Results

### Participant characteristics

Of 818 individuals who initiated the survey, 808 met the inclusion criteria and were incorporated into the final analysis. The final cohort consisted of 377 dentists (46.7%), 227 CFPs (28.1%), and 204 FMSs (25.2%). Females constituted 70.3% of the total sample. A statistically significant intergroup difference in sex distribution was observed (*p* < 0.001; Cramér’s V = 0.25). Certified family physicians demonstrated substantially greater professional experience than the other groups (median 11 [5–22] years vs. 3 [2–6] years for dentists and 4 [2–7] years for FMSs; *p* < 0.001). Detailed demographic data are presented in Table 1.

**Table 1:** Sociodemographic characteristics of study participants

Variable	Dentists (*n* = 377)	CFPs (*n* = 227)	FMSs (*n* = 204)	*p*-value
**Sex**,** n (%)**				
Female	307 (81.4)	125 (55.1)	136 (66.7)	< 0.001
Male	70 (18.6)	102 (44.9)	68 (33.3)	
**Age group**,** n (%)**				
≤ 29 years	286 (75.9)	53 (23.3)	118 (57.8)	< 0.001
≥ 30 years	91 (24.1)	174 (76.7)	86 (42.2)	
**Professional experience**,** median (IQR)**,** years**	3 (2–6)	11 (5–22)	4 (2–7)	< 0.001

*CFPs* certified family physicians, *FMSs* family medicine specialists and residents, *IQR* interquartile range. Different superscript letters denote statistically distinct subgroups after Bonferroni-corrected post-hoc analysis

### Knowledge of periodontal disease characteristics and clinical diagnosis

**Fig. 1:**
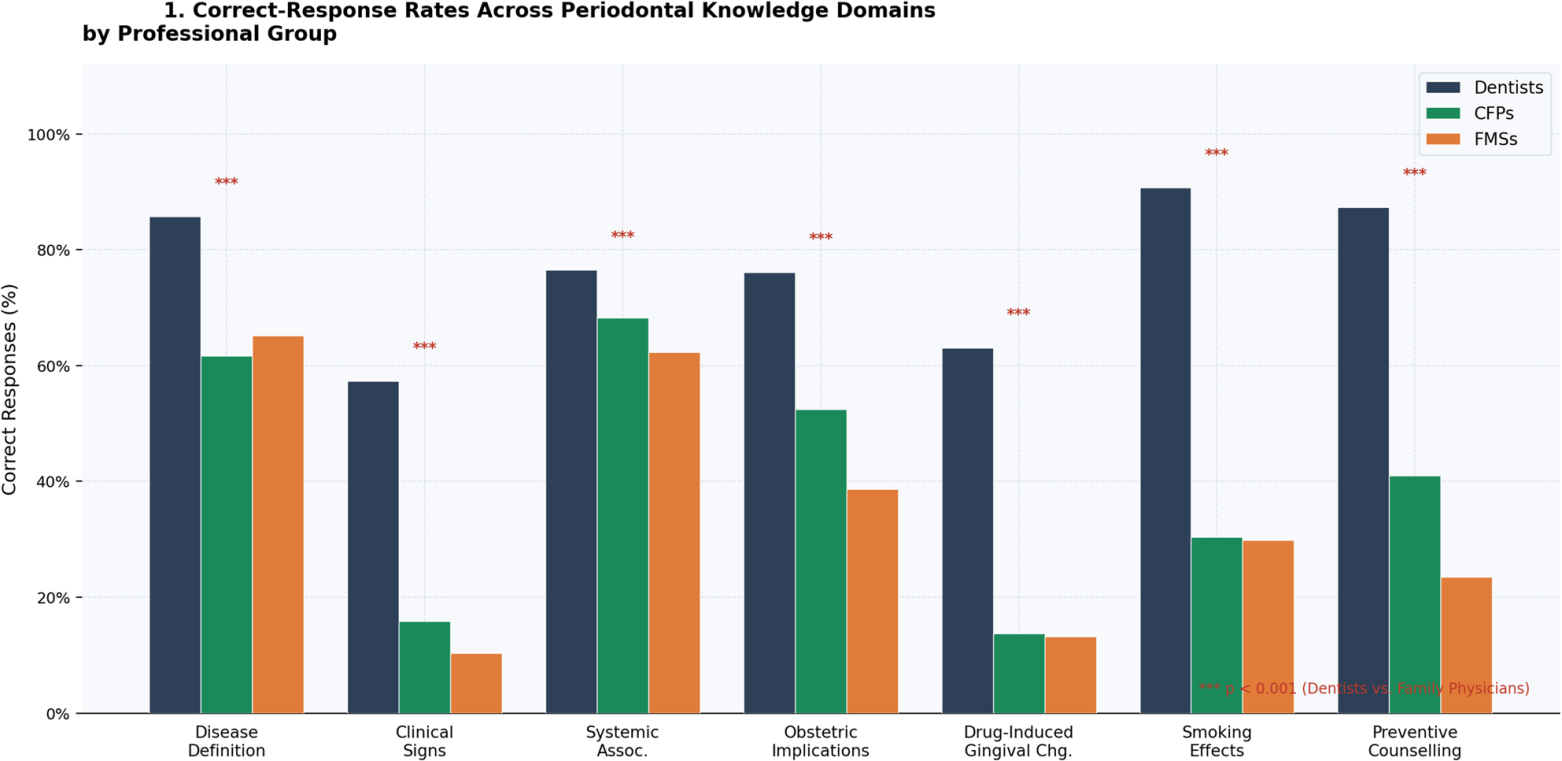
Correct-response rates across periodontal knowledge domains by professional group. Grouped bar chart comparing correct-response percentages in seven knowledge domains among dentists (*n* = 377), certified family physicians—CFPs (*n* = 227), and family medicine specialists/residents—FMSs (*n* = 204). *** *p* < 0.001 for dentists versus each family physician group (Bonferroni-corrected post-hoc analysis)

The capacity to correctly define periodontal disease was significantly higher among dentists (85.7%) than CFPs (61.7%) or FMSs (65.2%), with no significant difference between the two family physician subgroups (Cramér’s V = 0.25). A particularly notable difference was identified in recognition of pathognomonic clinical signs: only 15.9% of CFPs and 10.3% of FMSs could correctly identify all cardinal features—gingival bleeding, tooth mobility, and gingival recession—compared with 57.3% of dentists (χ² = 176.1, *p* < 0.001; Cramér’s V = 0.47, indicating a large effect). Among family physicians, 69.2% of CFPs and 71.6% of FMSs selected toothache—which is not a typical clinical manifestation of this often pain-free condition—as a primary symptom (*p* < 0.001; Cramér’s V = 0.38). These differences may in part reflect the distinct scope and focus of periodontal training within dental versus medical curricula, rather than a fundamental gap in clinical reasoning capacity. After adjustment for age group, sex, and years of clinical experience in multivariable logistic regression models, professional group remained a significant independent predictor of correct responses across all primary knowledge domains (Table S1). A cross-domain comparison of correct-response rates is illustrated in Fig. 1; item-level data are presented in Table 2.

**Table 2:** Knowledge of periodontal disease characteristics and clinical features

Survey item	Dentists *n* (%)	CFPs *n* (%)	FMSs *n* (%)	*p*	Cramér’s V
1. Correct definition of periodontal disease	323 (85.7)^a^	140 (61.7)^b^	133 (65.2)^b^	< 0.001	0.25
2. All correct clinical signs identified (bleeding, mobility, recession)	216 (57.3)^a^	36 (15.9)^b^	21 (10.3)^b^	< 0.001	0.47
3. Toothache incorrectly selected as primary sign (error rate)	123 (32.6)^b^	157 (69.2)^a^	146 (71.6)^a^	< 0.001	0.38
4. Periodontitis can cause loss of all teeth	353 (93.6)^a^	151 (66.5)^b^	133 (65.2)^b^	< 0.001	0.34
5. Gingival bleeding is an important clinical sign	354 (93.9)^a^	195 (85.9)^b^	179 (87.7)^ab^	0.003	0.12
6. Disease can progress without subjective symptoms	325 (86.2)	196 (86.3)	179 (87.7)	0.864	0.02
7. Radiographic examination is important in diagnosis	360 (95.5)^a^	103 (45.4)^b^	78 (38.2)^b^	< 0.001	0.57

Different superscript letters denote statistically distinct subgroups after Bonferroni-corrected post-hoc analysis (*p* < 0.05). Shared superscripts indicate no statistically significant difference

### Knowledge of periodontal–systemic disease associations

The bidirectional relationship between diabetes mellitus and periodontitis was broadly recognized, though significantly less so among family physicians (dentists 97.6%, CFPs 88.5%, FMSs 90.7%; *p* < 0.001; Cramér’s V = 0.16). Knowledge of the reverse relationship—that periodontitis actively complicates glycemic regulation—was substantially lower (dentists 79.3%, CFPs 68.7%, FMSs 54.4%; *p* < 0.001; Cramér’s V = 0.22). Regarding broader systemic correlations including coronary artery disease, cerebrovascular disorders, rheumatoid arthritis, and peripheral artery diseases, family physicians selected the ‘I do not know’ response at a significantly higher frequency (42.7% for both CFP and FMS groups) compared with dentists (3.2%; *p* < 0.001; Cramér’s V = 0.46). Results are presented in Table 3.

**Table 3:** Knowledge of periodontal–systemic disease associations

Survey item	Dentists *n* (%)	CFPs *n* (%)	FMSs *n* (%)	*p*	Cramér’s V
8. Diabetes mellitus is a risk factor for periodontitis	368 (97.6)^a^	201 (88.5)^b^	185 (90.7)^b^	< 0.001	0.16
9. Periodontitis complicates glycemic control in diabetics	299 (79.3)^a^	156 (68.7)^b^	111 (54.4)^c^	< 0.001	0.22
10. Periodontal treatment positively affects diabetes prognosis	278 (73.7)^a^	148 (65.2)^ab^	113 (55.4)^b^	< 0.001	0.16
11. Correctly identified ≥ 3 associated systemic diseases	362 (96.1)^a^	190 (83.8)^b^	168 (82.4)^b^	< 0.001	0.19
12. ‘Don’t know’ response ≥ 1 systemic association item	12 (3.2)^b^	97 (42.7)^a^	87 (42.7)^a^	< 0.001	0.46

Different superscript letters denote statistically distinct subgroups after Bonferroni-corrected post-hoc analysis (*p* < 0.05)

### Knowledge of obstetric implications and drug-induced gingival changes

Awareness of adverse pregnancy outcomes linked to periodontitis was substantially lower among family physicians: 52.5% of CFPs and 38.7% of FMSs identified at least one such outcome, versus 76.1% of dentists (*p* < 0.001; Cramér’s V = 0.32), with an additional significant gradient between CFP and FMS subgroups (*p* = 0.010). The ‘calcium transfer’ myth was endorsed by 59.5% of CFPs and 60.3% of FMSs versus only 10.9% of dentists (*p* < 0.001; Cramér’s V = 0.51). Only 13.7% of CFPs and 13.2% of FMSs could identify all three drug classes causing gingival enlargement (immunosuppressants, calcium channel blockers, anticonvulsants) compared with 63.1% of dentists (*p* < 0.001; Cramér’s V = 0.51). Data are presented in Table 4.

**Table 4:** Knowledge of obstetric implications and drug-induced gingival changes

Survey item	Dentists *n* (%)	CFPs *n* (%)	FMSs *n* (%)	*p*	Cramér’s V
13. Identified ≥ 1 adverse pregnancy outcome linked to periodontitis	287 (76.1)^a^	119 (52.5)^b^	79 (38.7)^c^	< 0.001	0.32
14. Periodontal conditions worsen during pregnancy	373 (98.9)^a^	178 (78.4)^b^	171 (83.8)^b^	< 0.001	0.30
15. Correctly rejected the ‘calcium transfer’ myth	336 (89.1)^a^	92 (40.5)^b^	81 (39.7)^b^	< 0.001	0.51
16. 2nd trimester is safest period for dental procedures	361 (95.8)^a^	119 (52.4)^b^	108 (52.9)^b^	< 0.001	0.48
17. Correctly identified all 3 drug classes causing gingival enlargement	238 (63.1)^a^	31 (13.7)^b^	27 (13.2)^b^	< 0.001	0.51

Different superscript letters denote statistically distinct subgroups after Bonferroni-corrected post-hoc analysis (*p* < 0.05)

### Clinical attitudes: preventive counselling, tobacco awareness, and referral behavior

**Fig. 2:**
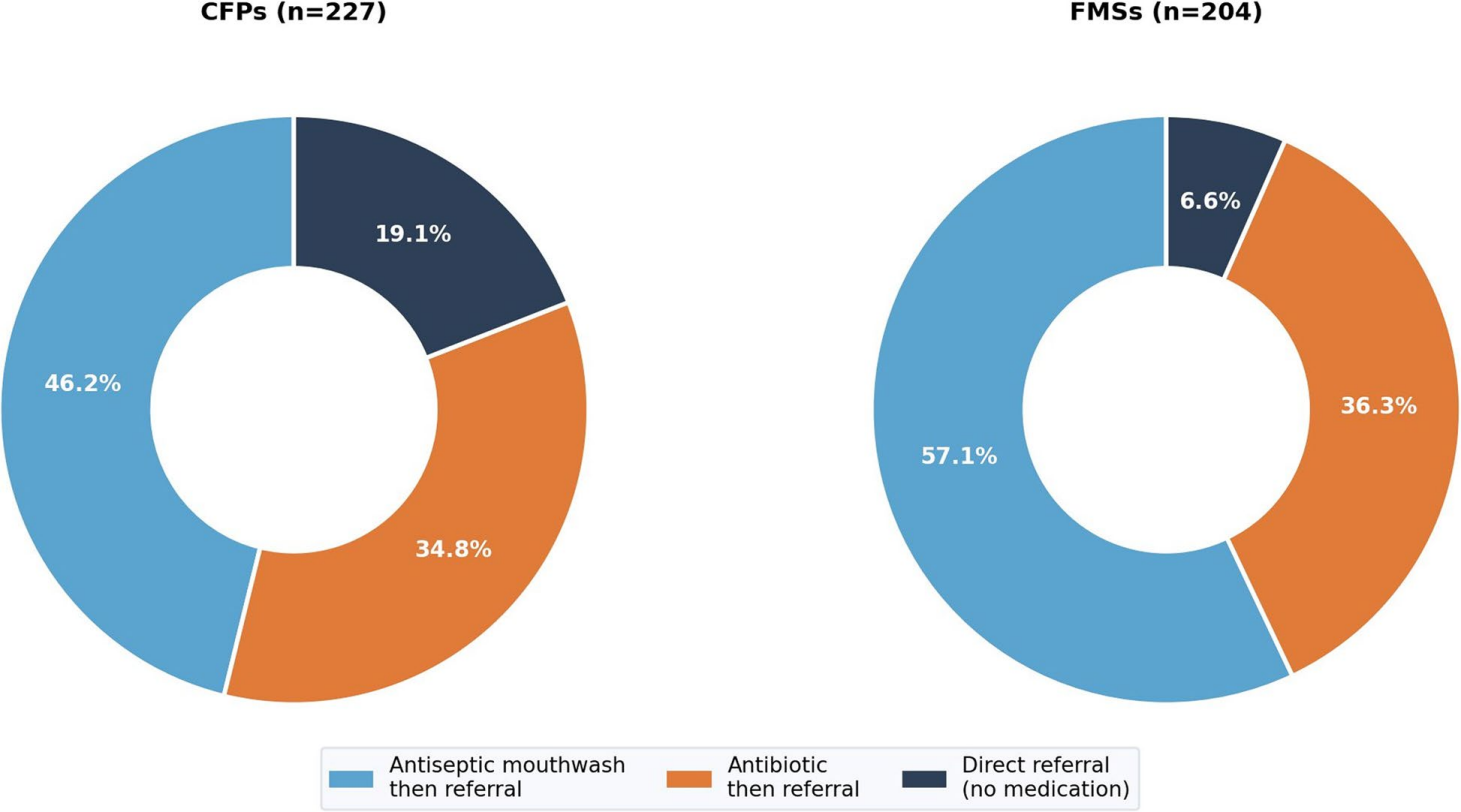
Management behavior for suspected dental infection among family physicians. Donut charts showing the proportional distribution of referral behaviors in CFPs (left) and FMSs (right). Direct referral without prior medication was significantly more frequent in CFPs than FMSs (24.2% vs. 8.3%, *p* < 0.001)

Only 30.4% of CFPs and 29.9% of FMSs recognized that smoking suppresses gingival bleeding, compared with 90.7% of dentists (*p* < 0.001; Cramér’s V = 0.61). Oral hygiene counselling was provided routinely by 87.3% of dentists but only 41.0% of CFPs and 23.5% of FMSs (*p* < 0.001; Cramér’s V = 0.57). Direct referral for suspected dental infection without antibiotic or antiseptic prescription was observed in only 24.2% of CFPs and 8.3% of FMSs (*p* < 0.001), as illustrated in Fig. 2. Attitudinal data are presented in Table 5.

**Table 5:** Clinical attitudes: preventive counselling, tobacco awareness, and referral behavior

Survey item	Dentists *n* (%)	CFPs *n* (%)	FMSs *n* (%)	*p*	Cramér’s V
18. Smoking suppresses clinical signs of periodontal disease	342 (90.7)^a^	69 (30.4)^b^	61 (29.9)^b^	< 0.001	0.61
19. Routinely provides oral hygiene counselling to patients	329 (87.3)^a^	93 (41.0)^b^	48 (23.5)^c^	< 0.001	0.57
20. Direct referral without prior medication for suspected dental infection	N/A^a^	55 (24.2)^a^	17 (8.3)^b^	< 0.001	0.21
21. Self-assessed periodontal knowledge ‘good’ (7–10/10)	312 (82.8)^a^	38 (16.6)^b^	21 (10.3)^b^	< 0.001	0.69

N/A not applicable (item not relevant to dentist practice). Different superscript letters denote statistically distinct subgroups after Bonferroni-corrected post-hoc analysis (*p* < 0.05)

### Self-assessment and sources of periodontal knowledge

**Fig. 3:**
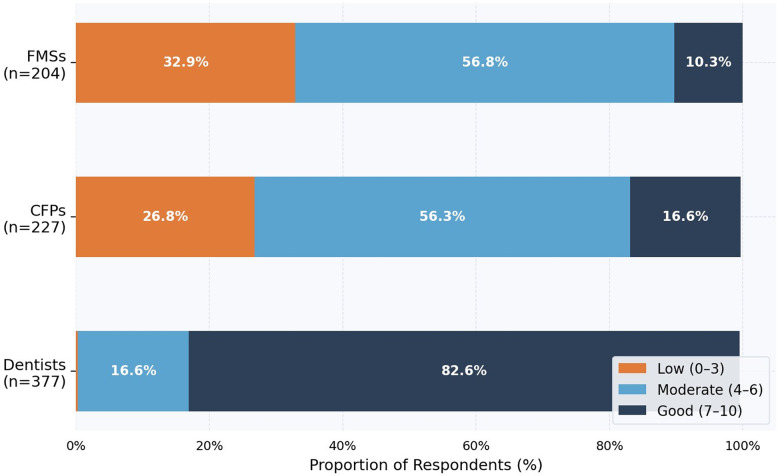
Self-assessment of periodontal knowledge (10-point visual numeric scale). Stacked horizontal bar chart showing the proportional distribution of self-rated periodontal knowledge. Low: 0–3; Moderate: 4–6; Good: 7–10. CFPs certified family physicians; FMSs family medicine specialists and residents

**Fig. 4:**
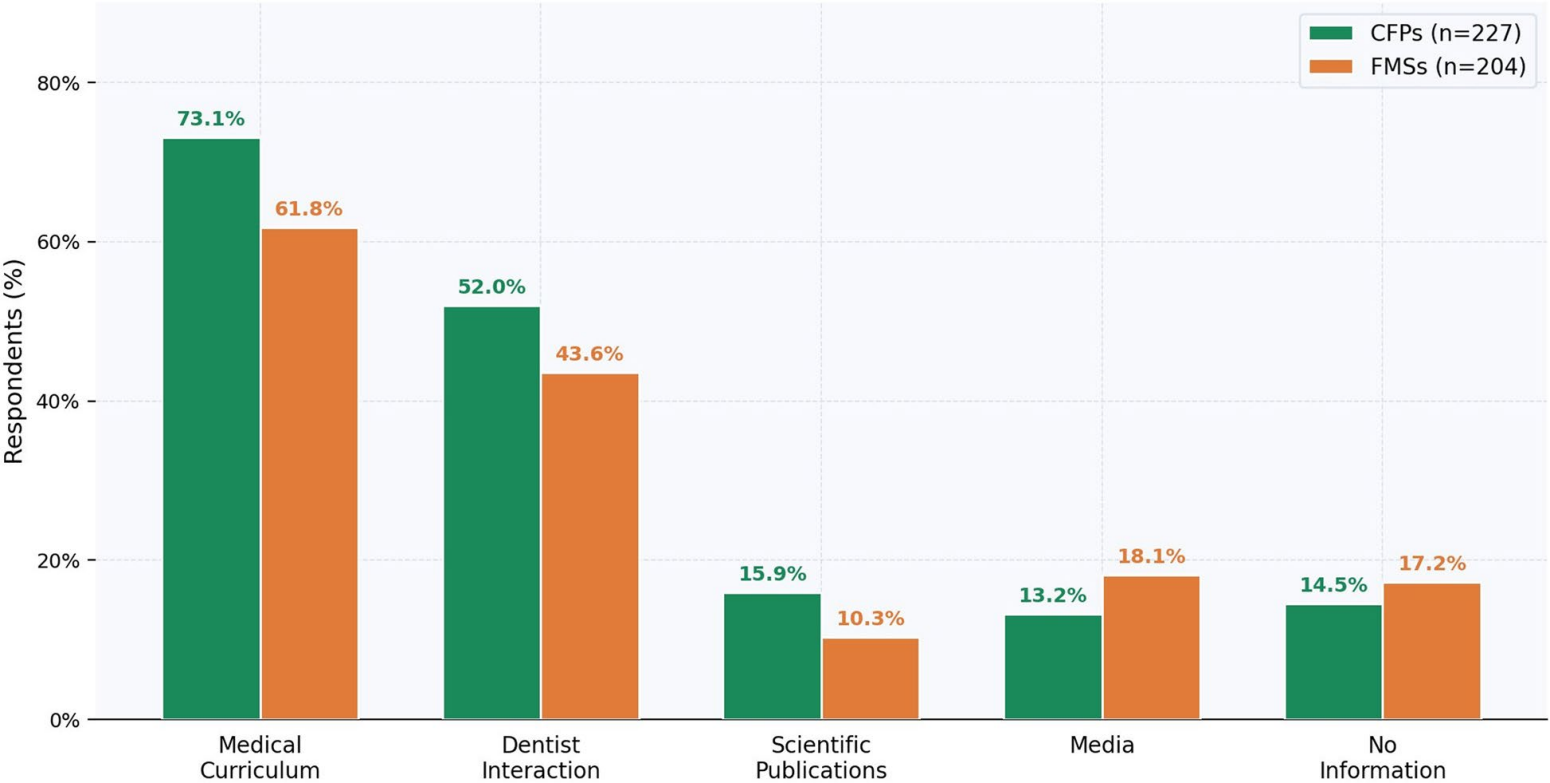
Sources of periodontal knowledge among family physicians. Grouped bar chart comparing information sources for CFPs and FMSs. Multiple responses were permitted per participant

Only 16.6% of CFPs and 10.3% of FMSs rated their periodontal knowledge as ‘good’ (7–10/10), compared with 82.8% of dentists (Fig. 3). Primary knowledge sources among family physicians were undergraduate medical curricula (CFPs 73.1%; FMSs 61.8%) and interactions with dentists (CFPs 52.0%; FMSs 43.6%); 14.5% of CFPs and 17.2% of FMSs reported no information source (Fig. 4).

### Personal oral hygiene practices and self-reported periodontal status

**Fig. 5:**
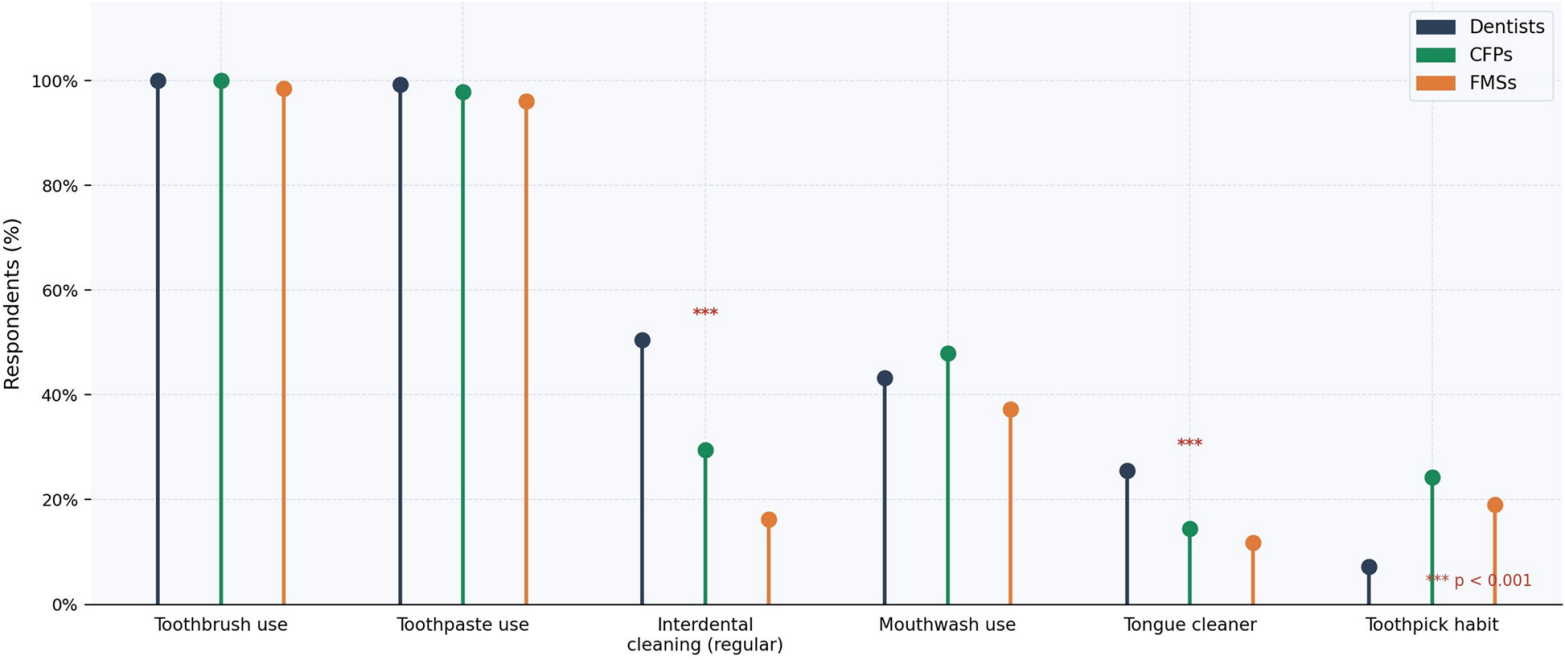
Personal oral hygiene practices by professional group. Lollipop chart comparing oral hygiene behaviors. *** *p* < 0.001 (Bonferroni-corrected)

**Fig. 6:**
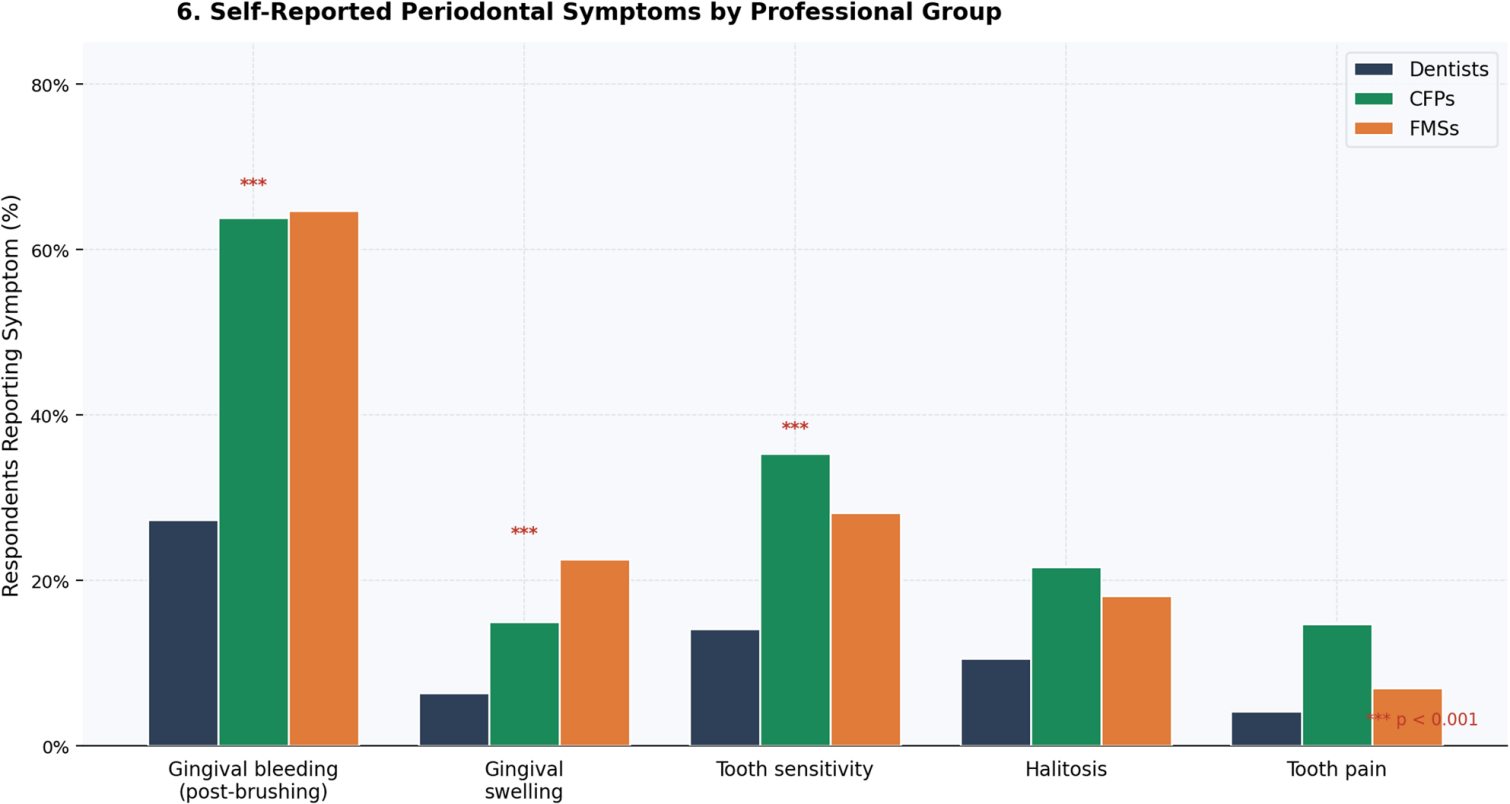
Self-reported periodontal symptoms by professional group. Grouped bar chart comparing prevalence of self-reported symptoms. *** *p* < 0.001

Regular use of interproximal cleaning aids was markedly less frequent among family physicians (CFPs 29.5%; FMSs 16.2%) than dentists (50.5%; *p* < 0.001). Self-reported gingival bleeding after brushing was substantially more prevalent in family physicians (CFPs 63.8%; FMSs 64.7%) than dentists (27.3%; *p* < 0.001). Oral hygiene practices and self-reported symptoms are depicted in Figs. 5 and 6, respectively.

## Discussion

This study analyzed the periodontal knowledge and clinical attitudes of family physicians across Turkey, employing dental practitioners as a professionally trained reference group. Our findings suggest that family physicians may harbor notable knowledge gaps regarding periodontal etiology, clinical diagnosis, and risk management, and that their current clinical attitudes appear to diverge from recommended interdisciplinary referral and preventive care practices (Fig. 1). It is important to emphasize that the knowledge gaps identified in this study reflect self-reported responses to a structured questionnaire and should not be equated with clinical incompetence. Family physicians operate within a different scope of practice than dentists; family physicians operate within a different scope of practice and professional domain, and the absence of detailed periodontal knowledge does not diminish their competence within their primary area of practice. Rather, these findings highlight an opportunity for cross-disciplinary education that could enhance the integrated management of patients with both oral and systemic conditions.

The finding that nearly 70% of family physicians perceived toothache as a primary clinical sign of periodontitis may reflect a fundamental gap in the clinical understanding of periodontal pathophysiology. The failure to appreciate the chronic and insidious nature of periodontitis—which typically progresses silently for years before causing pain—could potentially contribute to delayed referrals, often occurring only after irreversible attachment and bone destruction has already manifested [20]. This observation is consistent with comparable investigations conducted in France [16] and India [15], suggesting that gaps in periodontal clinical literacy among primary care practitioners may be a widespread phenomenon. Notably, the uniform recognition across all groups that periodontitis can progress without subjective symptoms (*p* = 0.864) implies an intellectual awareness that may not be successfully translated into appropriate clinical reasoning about diagnostic presentations. These differences persisted after adjustment for demographic confounders in our multivariable analyses, suggesting that they are not fully attributable to age or experience differences between the groups.

The observed tendency for antibiotic utilization among family physicians managing suspected dental infections is noteworthy (Fig. 2). The finding that over 75% of participating family physicians reported prescribing antibiotics or antiseptic mouthwashes prior to dental referral may reflect gaps in interdisciplinary referral education, with potential implications for antimicrobial resistance (AMR) [21]. This pattern may suggest a prevailing assumption that oral inflammatory conditions can be managed through systemic pharmacotherapy rather than definitive dental treatment. Addressing this behavior may benefit from both educational initiatives and the development of clearer interdisciplinary referral guidelines.

A substantial proportion of family physicians recognized the bidirectional relationship between diabetes and periodontitis. This awareness aligns with findings from Hong Kong [17] and appears higher than those reported from France [16]. However, the widespread lack of awareness regarding broader systemic associations—including coronary artery disease, rheumatoid arthritis, and adverse pregnancy outcomes is notable [4, 6, 22]. A meta-analysis by Borgnakke et al. demonstrated that periodontal treatment is associated with a clinically meaningful reduction in HbA1c in diabetic patients [23]. yet fewer than 56% of FMSs in our cohort recognized this relationship, representing a potential missed opportunity for integrated chronic disease management. Similarly, the endorsement of the ‘calcium transfer’ myth by approximately 60% of family physicians could potentially lead to inappropriate clinical reassurance and failure to refer pregnant patients at periodontal risk.

The tobacco masking effect—whereby smoking suppresses gingival bleeding and thereby conceals clinical signs of active periodontal inflammation—was recognized by only approximately 30% of family physicians. A practitioner unaware of this phenomenon may interpret the absence of bleeding in a smoking patient as indicative of good periodontal health, potentially leading to false reassurance and missed diagnostic opportunities [24]. Given that family physicians are primary prescribers of smoking cessation therapies, the integration of tobacco–periodontal interactions into routine consultations could represent an important area for continuing education.

Self-reported oral health data (Figs. 5 and 6) indicate that family physicians reported a disproportionately higher burden of periodontal symptoms than dentists, including higher rates of gingival bleeding and lower rates of interproximal cleaning. These observational associations are exploratory and should not be interpreted as evidence that personal hygiene practices causally influence professional behavior; however, they may provide context for understanding the knowledge-attitude-practice continuum, consistent with the ‘physician health–practice style’ hypothesis. The predominant reliance on undergraduate medical training as the primary knowledge source (Fig. 4), combined with the majority of family physicians self-rating their knowledge as ‘moderate’ or ‘low’ (Fig. 3), suggests potential receptivity to continuing education interventions [25].

These findings should be interpreted in light of several important methodological considerations. The convenience sampling strategy and social media–based distribution may have attracted respondents with higher-than-average digital literacy and potentially greater health awareness, which could bias results toward overestimation of actual knowledge levels among family physicians. If so, the true knowledge gap in the broader population may be even larger than observed. Furthermore, while our adjusted analyses suggest that demographic confounders do not fully account for the observed differences, residual confounding by unmeasured variables (e.g., practice setting, patient population, continuing education participation) cannot be excluded in a cross-sectional design.

### Limitations

Several limitations warrant consideration when interpreting these findings. First, the convenience sampling strategy via social media platforms introduces selection bias; practitioners who voluntarily responded to a social media-distributed survey may have higher baseline interest in oral health topics, potentially leading to overestimation of knowledge levels. Conversely, rural practitioners and those less active on social media may be underrepresented, limiting generalizability to the entire Turkish family physician population. Second, the exact denominator for calculating a traditional response rate is unavailable due to the open nature of social media distribution, precluding assessment of non-response bias. Third, the cross-sectional design precludes causal inferences; the observed associations between professional group and knowledge levels cannot establish temporal or causal relationships. Fourth, all data are self-reported, introducing the potential for social desirability bias, particularly regarding clinical behaviors such as referral practices and oral hygiene counselling frequency. Fifth, while multivariable regression was performed to adjust for measured confounders, residual confounding by unmeasured variables—including practice setting, patient volume, prior continuing education exposure, and geographic location—cannot be excluded. Sixth, the questionnaire instrument, while demonstrating acceptable internal consistency (KR-20 = 0.74), did not undergo comprehensive psychometric validation including test-retest reliability and confirmatory factor analysis. Seventh, the sample sizes achieved for the CFP and FMS groups fell below the a priori calculation, potentially reducing precision of effect estimates for smaller differences. Eighth, the concurrent COVID-19 pandemic during data collection may have influenced both recruitment patterns and respondent engagement. Notwithstanding these limitations, the large multi-group sample and the consistency of findings with comparable international studies provide a reasonable characterization of the periodontal knowledge landscape within Turkish primary care.

## Conclusions

This cross-sectional study suggests that family physicians in Turkey may have notable gaps in periodontal knowledge across multiple assessed domains, including disease recognition, systemic associations, obstetric implications, and referral practices. After adjustment for demographic confounders, professional group remained independently associated with knowledge outcomes, suggesting that these differences are not entirely attributable to age or experience variations between groups. While these findings should be interpreted with caution given the convenience sampling methodology and cross-sectional design, they are consistent with observations from comparable international studies.

Future research employing probability-based sampling and longitudinal or interventional designs would be valuable to confirm these findings and to evaluate whether structured educational programs could improve periodontal literacy among primary care providers. The development of interprofessional ‘Oral–Systemic Health’ educational modules and evidence-based referral guidelines merits consideration, pending confirmatory evidence from appropriately designed studies.

## Supplementary Information

Supplementary Material 1.

Supplementary Material 2.

## Data Availability

The anonymized dataset supporting the findings of this study is available from the corresponding author upon reasonable request. Aggregate data underlying all reported findings are presented in the tables and figures within this manuscript and its supplementary materials. Requests for the full anonymized dataset will be considered on an individual basis, subject to compliance with applicable data protection regulations.

## Abbreviations

AMR: Antimicrobial Resistance
aOR: Adjusted Odds Ratio
CFPs: Certified Family Physicians
CI: Confidence Interval
COPD: Chronic Obstructive Pulmonary Disease
EFP: European Federation of Periodontology
FDI: World Dental Federation (Fédération Dentaire Internationale)
FMSs: Family Medicine Specialists and residents
GBD: Global Burden of Disease
HbA1c: Glycated hemoglobin
IQR: Interquartile Range
KAP: Knowledge, Attitudes, Practice
KR-20: Kuder–Richardson Formula 20
NICE: National Institute for Health and Care Excellence
OR: Odds Ratio
SPSS: Statistical Package for the Social Sciences

## References

[1] Glick M, Williams DM, Kleinman DV, Vujicic M, Watt RG, Weyant RJ. A new definition for oral health developed by the FDI World Dental Federation opens the door to a universal definition of oral health. J Am Dent Assoc. 2016;147(12):915–7. 27886668 10.1016/j.adaj.2016.10.001

[2] Kassebaum NJ, Smith AGC, Bernabe E, Fleming TD, Reynolds AE, Vos T, et al. Global, regional, and national prevalence, incidence, and disability-adjusted life years for oral conditions for 195 countries, 1990–2015: a systematic analysis for the Global Burden of Diseases, Injuries, and Risk Factors. J Dent Res. 2017;96(4):380–7. 28792274 10.1177/0022034517693566 PMC5912207

[3] Kassebaum NJ, Bernabé E, Dahiya M, Bhandari B, Murray CJL, Marcenes W. Global burden of severe periodontitis in 1990–2010: a systematic review and meta-regression. J Dent Res. 2014;93(11):1045–53. 25261053 10.1177/0022034514552491 PMC4293771

[4] Chapple ILC, Mealey BL, Van Dyke TE, Bartold PM, Dommisch H, Eickholz P, et al. Periodontal health and gingival diseases and conditions on an intact and a reduced periodontium: consensus report of workgroup 1 of the 2017 World Workshop on the Classification of Periodontal and Peri-Implant Diseases and Conditions. J Periodontol. 2018;89(Suppl 1):S74–84. 29926944 10.1002/JPER.17-0719

[5] Papapanou PN, Sanz M, Buduneli N, Dietrich T, Feres M, Fine DH et al. Periodontitis: Consensus report of workgroup 2 of the 2017 World Workshop on the Classification of Periodontal and Peri-Implant Diseases and Conditions.J Periodontol. 2018;89 Suppl 1:S173–82. 10.1002/JPER.17-0721 29926951

[6] Tse SY. Diabetes mellitus and periodontal disease: awareness and practice among doctors working in public general out-patient clinics in Kowloon West Cluster of Hong Kong. BMC Fam Pract. 2018;19(1):199. 30558542 10.1186/s12875-018-0887-2 PMC6297978

[7] Marcenes W, Kassebaum NJ, Bernabé E, et al. Global burden of oral conditions in 1990–2010: a systematic analysis. J Dent Res. 2013;92(7):592–7. 23720570 10.1177/0022034513490168 PMC4484374

[8] Sanz M, Herrera D, Kebschull M, et al. Treatment of stage I–III periodontitis—The EFP S3 level clinical practice guideline. J Clin Periodontol. 2020;47(S22):4–60. 32383274 10.1111/jcpe.13290 PMC7891343

[9] Sanz M, Ceriello A, Buysschaert M, et al. Scientific evidence on the links between periodontal diseases and diabetes. J Clin Periodontol. 2018;45(2):138–49. 29280174 10.1111/jcpe.12808

[10] Peres MA, Macpherson LMD, Weyant RJ, et al. Oral diseases: a global public health challenge. Lancet. 2019;394:249–60. 31327369 10.1016/S0140-6736(19)31146-8

[11] Al Agili DE, Farsi D, Mirza L, Farsi N. Primary care physicians and oral health care: competency gaps and opportunities for training. BMC Oral Health. 2025;25(1):159. 41310640 10.1186/s12903-025-07180-y PMC12750821

[12] Watt RG, Daly B, Allison P, Macpherson LMD, Venturelli R, Listl S et al. Ending the neglect of global oral health: time for radical action. Lancet 2019 Jul 20–26;394(10194):261–72. 10.1016/S0140-6736(19)31133-X 31327370

[13] Choi SE, Simon L, Barrow JR, Palmer N, Basu S, Phillips RS. Dental practice integration into primary care: A microsimulation of financial implications for practices. Int J Environ Res Public Health. 2020;17(6):2154. 10.3390/ijerph17062154 PMC7175120 32213882

[14] Hancı ST, Güler E, Hancı Ö. Examination of the knowledge levels of family medicine residents in Turkey about oral and dental health. Oral Health Prev Dent. 2024;22:b5656296. 39105313 10.3290/j.ohpd.b5656296 PMC11619888

[15] Nagarakanti S, Epari V, Athuluru D. Knowledge, attitude, and practice of medical doctors towards periodontal disease. J Indian Soc Periodontol. 2013;17(1):137–9. 23633790 10.4103/0972-124X.107491 PMC3636935

[16] Boutigny H, Dufour T, Boschin F, Delcourt-Debruyne E, Tenenbaum H, Borenstein P. Assessment of knowledge, attitudes and practices concerning oral health among general medical practitioners. J Periodontal Res. 2008;43(4):395–400.

[17] Lim LP, Davies WIR, Yuen KW, Ma MH. Physicians’ knowledge about periodontal disease-diabetes relationships in Hong Kong. J Clin Periodontol. 2009;36:1013–20.

[18] Bahramian H, Nourizadeh M. Knowledge, attitude and practices of family physicians regarding oral health in Iran. J Dent (Shiraz). 2013;10(5):432–8.

[19] Wu Y, Dong G, Chen Y, Wang Z, Meng H. Knowledge and attitudes of endocrinologists and dentists regarding the relationship between diabetes and periodontitis. Eur J Dent Educ. 2017;21(1):60–7.

[20] Tonetti MS, Jepsen S, Jin L, Otomo-Corgel J. Impact of the global burden of periodontal diseases on health, nutrition and wellbeing of mankind: a call for global action. J Clin Periodontol. 2017;44(5):456–62. 28419559 10.1111/jcpe.12732

[21] NICE. Antimicrobial stewardship: changing risk-related behaviours in the general population. NICE guideline NG63. London: NICE; 2017.

[22] Jepsen S, Caton JG, Albandar JM, et al. Periodontal manifestations of systemic diseases and developmental and acquired conditions: Consensus report of workgroup 3 of the 2017 World Workshop on the Classification of Periodontal and Peri-Implant Diseases and Conditions. J Clin Periodontol. 2018;45(Suppl 20):S219–29. 29926500 10.1111/jcpe.12951

[23] Borgnakke WS, Ylöstalo PV, Taylor GW, Genco RJ. Effect of periodontal disease on diabetes: systematic review of epidemiologic observational evidence. J Periodontol. 2013;84(Suppl 4):S135–52. 23631574 10.1902/jop.2013.1340013

[24] Buduneli N, Scott DA. Tobacco-induced suppression of the vascular response to dental plaque. Mol Oral Microbiol. 2018;33(4):271–82. 29768735 10.1111/omi.12228 PMC8246627

[25] DeLong LA, Burkhard MJ, Creasey JL, Lanning SK. Oral health training in U.S. medical schools: a national survey. J Dent Educ. 2017;81(4):416–22.

