# Supplementary material for: Periodontal knowledge and clinical attitudes of family physicians in Turkey: a cross-sectional survey
Source: BMC Prim Care. 2026 Apr 22;27:224. doi: 10.1186/s12875-026-03324-3 (PMC13238091; doi:10.1186/s12875-026-03324-3)
Supplement: Supplementary file 1 — Supplementary Material 1. [file 12875_2026_3324_MOESM1_ESM.pdf]

Personal Information:

1. Age:
☐ ≤29
☐ 30–39
☐ 40–49
☐ 50–59
☐ ≥60

2. Gender:
☐ Female
☐ Male

3. Marital Status:
☐ Married
☐ Single

4. Educational Status:
☐ Contracted / Certified Family Physician
☐ Family Medicine Resident
☐ Dentist
☐ Other: ___________

5. Year of Graduation: _______________________

6. How long have you been working at a Family Health Center? (in months)

…………………

(Family medicine residents are kindly requested not to answer.)

Questions About Knowledge and Attitudes Toward Periodontal Health and Disease:

1. What is periodontal disease (periodontitis)?

☐ Infection of the tooth
☐ Infection of the gums
☐ Inflammatory disease of the supporting tissues of the tooth (periodontal ligament, cementum and alveolar bone)
☐ Infection of the supporting tissues of the tooth
☐ I do not know

---

2. What are the clinical signs of periodontitis?
(You may select more than one option)

☐ Tooth pain
☐ Bleeding gums
☐ Tooth mobility
☐ Gingival recession
☐ I do not know

---

3. “Periodontitis may lead to the loss of all teeth in the mouth.”

☐ True
☐ False
☐ I do not know

---

4. “Gingival bleeding is an important sign of periodontal disease.”

☐ True
☐ False
☐ I do not know

---

5. “Periodontal disease may progress for a long time without causing any subjective symptoms.”

☐ True
☐ False
☐ I do not know

---

6. “Radiographic examination plays an important role in the diagnosis of periodontitis.”

☐ True
☐ False
☐ I do not know

---

7. Which factors play a role in the development of periodontal disease?
(You may select more than one option)

☐ Poor oral hygiene
☐ Dental caries
☐ Dental calculus
☐ Genetic factors
☐ Pregnancy

☐ Stress
☐ None
☐ I do not know

---

8. Which oral problems may be caused by periodontitis?
(You may select more than one option)

☐ Tooth mobility
☐ Tooth loss
☐ Dental caries
☐ Tooth pain
☐ Facial swelling
☐ Tooth sensitivity
☐ Halitosis (bad breath)
☐ None
☐ I do not know

---

9. "Diabetes mellitus is a risk factor for the development of periodontitis."

☐ True
☐ False
☐ I do not know

---

10. "Periodontitis may make glycemic control more difficult in diabetic individuals."

☐ True
☐ False
☐ I do not know

---

11. "Treatment of periodontal disease contributes positively to the prognosis of diabetes mellitus."

☐ True
☐ False
☐ I do not know

---

12. Which systemic diseases are associated with periodontal disease?
(You may select more than one option)

☐ Coronary artery diseases
☐ Cerebrovascular diseases
☐ Peripheral artery diseases
☐ Diabetes mellitus
☐ Rheumatoid arthritis
☐ None
☐ I do not know

---

13. Which of the following may occur in pregnant individuals with periodontal disease?
(You may select more than one option)

☐ Risk of low birth weight
☐ Risk of preterm birth
☐ Low risk
☐ None
☐ I do not know

---

14. "Existing periodontal conditions may worsen during pregnancy and severe inflammatory gingival enlargements may occur."

☐ True
☐ False
☐ I do not know

---

15. "Tooth loss during pregnancy occurs because minerals from the mother's teeth are transferred to the baby."

☐ True
☐ False
☐ I do not know

---

16. In which trimester is it most appropriate for pregnant individuals to undergo dental procedures?

☐ First trimester
☐ Second trimester
☐ Third trimester

---

17. Which drugs may cause gingival enlargement?
(You may select more than one option)

☐ Immunosuppressants
☐ Calcium channel blockers
☐ Anticonvulsants
☐ None
☐ I do not know

---

18. Which of the following statements about smoking are correct?
(You may select more than one option)

☐ Smoking cessation causes periodontal disease
☐ Smoking suppresses clinical signs (bleeding) of periodontal disease
☐ Smoking cessation positively affects periodontal treatment prognosis
☐ Smoking more than one pack per day is a risk factor for implant surgery
☐ None
☐ I do not know

---

19. "Periodontal diseases may also occur around dental implants."

☐ True
☐ False
☐ I do not know

---

20. "Dental implants can easily be placed to replace teeth lost due to periodontal disease."

☐ True
☐ False
☐ I do not know

---

21. What would you do for a patient presenting with suspected dental infection?
(You may select more than one option)

☐ I prescribe antiseptic mouthwash
☐ I prescribe antibiotics
☐ I prescribe antiseptic mouthwash and refer to a dentist
☐ I prescribe antibiotics and refer to a dentist
☐ I refer to a dentist without prescribing medication

---

22. “In the treatment of periodontal disease, the timing of antibiotic use has no importance when antibiotics are required.”

☐ True
☐ False
☐ I do not know

---

23. Do you give recommendations or reminders about oral hygiene to your patients?

☐ I do not give recommendations
☐ I sometimes give recommendations
☐ I usually give recommendations

---

24. “In preventing periodontal and other dental diseases, the content of toothpaste is more important than tooth brushing.”

☐ True
☐ False
☐ I do not know

---

25. How would you evaluate your knowledge about periodontal health and diseases?

0 1 2 3 4 5 6 7 8 9 10

(0 = No knowledge, 5 = Moderate knowledge, 10 = Sufficient knowledge)

---

26. What is the source of your knowledge about periodontal health and disease?
(You may select more than one option)

☐ Medical education
☐ Communication with dentists
☐ Scientific articles and books
☐ Media (television, internet)
☐ I have not received any information

Personal Oral Care and Habits:

1. What do you use for your personal oral care?
(You may select more than one option)

☐ Toothbrush
☐ Toothpaste
☐ Interdental brush and/or dental floss
☐ Tongue cleaner
☐ Mouthwash
☐ Oral irrigator
☐ Toothpick
☐ Miswak
☐ Other: ___________

---

2. How often do you brush your teeth?

☐ At least twice a day
☐ At least once a day
☐ At least three times a week
☐ Whenever I remember
☐ I do not brush
☐ Other: ___________

---

3. How often and with what do you clean between your teeth?

☐ I do not clean between my teeth
☐ I use toothpicks
☐ I regularly use interdental brushes or dental floss
☐ I rarely use interdental brushes or dental floss
☐ Other: ___________

---

4. Do your gums bleed after brushing?

☐ Frequently
☐ Rarely
☐ During brushing or spontaneously
☐ No bleeding

---

5. Do you have swelling in your gums?

☐ Widespread swelling
☐ Swelling in some areas
☐ No swelling
☐ I do not know

---

6. Do you have any complaints related to your mouth or teeth?
(You may select more than one option)

☐ No complaints
☐ Bleeding gums
☐ Difficulty chewing
☐ Appearance of teeth
☐ Speech problems
☐ Tooth sensitivity
☐ Bad breath
☐ Taste disturbance
☐ Toothache
☐ Other: ___________

---

7. Do you regularly visit a dentist?

☐ I have never visited a dentist
☐ I visit only when I have pain
☐ I sometimes go for check-ups
☐ I go for regular check-ups at least once a year

---

8. Are you informed about your periodontal health?

☐ I learned during routine check-ups that I am periodontally healthy
☐ I learned that I had periodontal disease but did not receive treatment
☐ I learned that I had periodontal disease and received treatment
☐ I went for a check-up but do not have sufficient knowledge
☐ I did not go for a check-up and do not have knowledge

---

9. Do you smoke?

☐ I do not smoke
☐ <10 cigarettes per day
☐ <1 pack per day
☐ >1 pack per day

---

10. Where did you learn your personal oral care practices?
(You may select more than one option)

☐ During medical education
☐ From a dentist
☐ Media (television, internet)
☐ I did not learn it anywhere
☐ Other: ___________
