# Supplementary material for: Periodontal knowledge and clinical attitudes of family physicians in Turkey: a cross-sectional survey
Source: BMC Prim Care. 2026 Apr 22;27:224. doi: 10.1186/s12875-026-03324-3 (PMC13238091; doi:10.1186/s12875-026-03324-3)
Supplement: Supplementary file 2 — Supplementary Material 2. [file 12875_2026_3324_MOESM2_ESM.docx]

**Supplementary Table S1.** Multivariable logistic regression analysis: adjusted odds ratios for correct responses across key periodontal knowledge and attitude domains by professional group

Binary logistic regression models were constructed for each outcome variable (1 = correct response, 0 = incorrect). Dentists served as the reference group. Professional group was entered as the primary independent variable, with age group (≤ 29 vs. ≥ 30 years), sex (male vs. female), and years of clinical experience (≤ 5 vs. > 5 years) included as covariates. Odds ratios < 1 indicate lower odds of a correct response relative to the dentist reference group.

| **Outcome variable** | **Group** | **Crude OR (95% CI)** | ***p*** | **aOR (95% CI)** | ***p*** |
| --- | --- | --- | --- | --- | --- |
| **Correct identification of all clinical signs** | Dentists | Ref. | — | Ref. | — |
|  | CFPs | 0.14 (0.09–0.21) | **< 0.001** | 0.17 (0.11–0.26) | **< 0.001** |
|  | FMSs | 0.09 (0.05–0.14) | **< 0.001** | 0.09 (0.06–0.15) | **< 0.001** |
| **Correct definition of periodontal disease** | Dentists | Ref. | — | Ref. | — |
|  | CFPs | 0.27 (0.18–0.40) | **< 0.001** | 0.31 (0.20–0.48) | **< 0.001** |
|  | FMSs | 0.31 (0.21–0.47) | **< 0.001** | 0.32 (0.21–0.49) | **< 0.001** |
| **Knowledge of ≥ 1 systemic disease association** | Dentists | Ref. | — | Ref. | — |
|  | CFPs | 0.21 (0.11–0.40) | **< 0.001** | 0.23 (0.12–0.45) | **< 0.001** |
|  | FMSs | 0.19 (0.10–0.36) | **< 0.001** | 0.21 (0.11–0.40) | **< 0.001** |
| **Awareness of adverse pregnancy outcomes** | Dentists | Ref. | — | Ref. | — |
|  | CFPs | 0.35 (0.24–0.49) | **< 0.001** | 0.38 (0.26–0.57) | **< 0.001** |
|  | FMSs | 0.20 (0.14–0.29) | **< 0.001** | 0.20 (0.14–0.30) | **< 0.001** |
| **Identification of all 3 drug classes causing gingival enlargement** | Dentists | Ref. | — | Ref. | — |
|  | CFPs | 0.09 (0.06–0.14) | **< 0.001** | 0.13 (0.08–0.21) | **< 0.001** |
|  | FMSs | 0.09 (0.06–0.14) | **< 0.001** | 0.09 (0.06–0.15) | **< 0.001** |
| **Recognition of smoking masking effect on gingival bleeding** | Dentists | Ref. | — | Ref. | — |
|  | CFPs | 0.04 (0.03–0.07) | **< 0.001** | 0.05 (0.03–0.09) | **< 0.001** |
|  | FMSs | 0.04 (0.03–0.07) | **< 0.001** | 0.04 (0.03–0.07) | **< 0.001** |
| **Routine oral hygiene counselling to patients** | Dentists | Ref. | — | Ref. | — |
|  | CFPs | 0.10 (0.07–0.15) | **< 0.001** | 0.10 (0.07–0.16) | **< 0.001** |
|  | FMSs | 0.04 (0.03–0.07) | **< 0.001** | 0.05 (0.03–0.07) | **< 0.001** |

**Supplementary Table S1b.** Covariate effects within each adjusted logistic regression model

| **Outcome model** | **Covariate** | **aOR (95% CI)** | ***p*** |
| --- | --- | --- | --- |
| **Correct identification of all clinical signs** | Age group (≥ 30 vs. ≤ 29 years) | 0.71 (0.37–1.37) | 0.311 |
|  | Sex (male vs. female) | 0.76 (0.51–1.13) | 0.170 |
|  | Experience (> 5 vs. ≤ 5 years) | 1.19 (0.65–2.21) | 0.572 |
| **Correct definition of periodontal disease** | Age group (≥ 30 vs. ≤ 29 years) | 0.93 (0.50–1.72) | 0.813 |
|  | Sex (male vs. female) | 0.95 (0.66–1.36) | 0.773 |
|  | Experience (> 5 vs. ≤ 5 years) | 0.79 (0.44–1.42) | 0.431 |
| **Knowledge of ≥ 1 systemic disease association** | Age group (≥ 30 vs. ≤ 29 years) | 0.65 (0.29–1.46) | 0.296 |
|  | Sex (male vs. female) | 0.77 (0.47–1.26) | 0.296 |
|  | Experience (> 5 vs. ≤ 5 years) | 1.73 (0.78–3.81) | 0.176 |
| **Awareness of adverse pregnancy outcomes** | Age group (≥ 30 vs. ≤ 29 years) | 1.30 (0.73–2.30) | 0.370 |
|  | Sex (male vs. female) | 0.55 (0.40–0.78) | **< 0.001** |
|  | Experience (> 5 vs. ≤ 5 years) | 0.80 (0.47–1.38) | 0.428 |
| **Identification of all 3 drug classes causing gingival enlargement** | Age group (≥ 30 vs. ≤ 29 years) | 0.90 (0.48–1.69) | 0.752 |
|  | Sex (male vs. female) | 0.55 (0.36–0.83) | **0.004** |
|  | Experience (> 5 vs. ≤ 5 years) | 0.60 (0.33–1.09) | 0.091 |
| **Recognition of smoking masking effect on gingival bleeding** | Age group (≥ 30 vs. ≤ 29 years) | 1.02 (0.53–1.96) | 0.950 |
|  | Sex (male vs. female) | 0.88 (0.59–1.30) | 0.510 |
|  | Experience (> 5 vs. ≤ 5 years) | 0.68 (0.36–1.28) | 0.236 |
| **Routine oral hygiene counselling to patients** | Age group (≥ 30 vs. ≤ 29 years) | 1.05 (0.54–2.04) | 0.886 |
|  | Sex (male vs. female) | 0.69 (0.47–1.00) | 0.053 |
|  | Experience (> 5 vs. ≤ 5 years) | 1.09 (0.58–2.06) | 0.783 |

*aOR = adjusted odds ratio; CI = confidence interval; CFPs = certified family physicians; FMSs = family medicine specialists and residents; Ref. = reference category.*

*All models adjusted for age group, sex, and years of clinical experience. Dentists served as the reference group in all models.*

*OR < 1.0 indicates lower odds of correct response compared with dentists. Bold p-values indicate statistical significance at the α = 0.05 level.*

*The consistency between crude and adjusted ORs across all outcomes indicates that the observed knowledge differences between professional groups are not attributable to confounding by age, sex, or clinical experience.*
